# Supplementary material for: Functional anatomy of zinc finger antiviral protein complexes
Source: Nat Commun. 2024 Dec 30;15:10834. doi: 10.1038/s41467-024-55192-z (PMC11685948; doi:10.1038/s41467-024-55192-z)
Supplement: Supplementary file 1 — Supplementary Information [file 41467_2024_55192_MOESM1_ESM.pdf]

Supplementary Information

Functional anatomy of Zinc Finger Antiviral Protein complexes

Jennifer A. Bohn, Jennifer L. Meagher, Matthew A. Takata, Daniel Gonçalves-Carneiro, Zoe C. Yeoh, Melanie D. Ohi, Janet L. Smith, Paul D. Bieniasz

Supplementary Figures 1 - 6
Supplementary Tables 1, 2

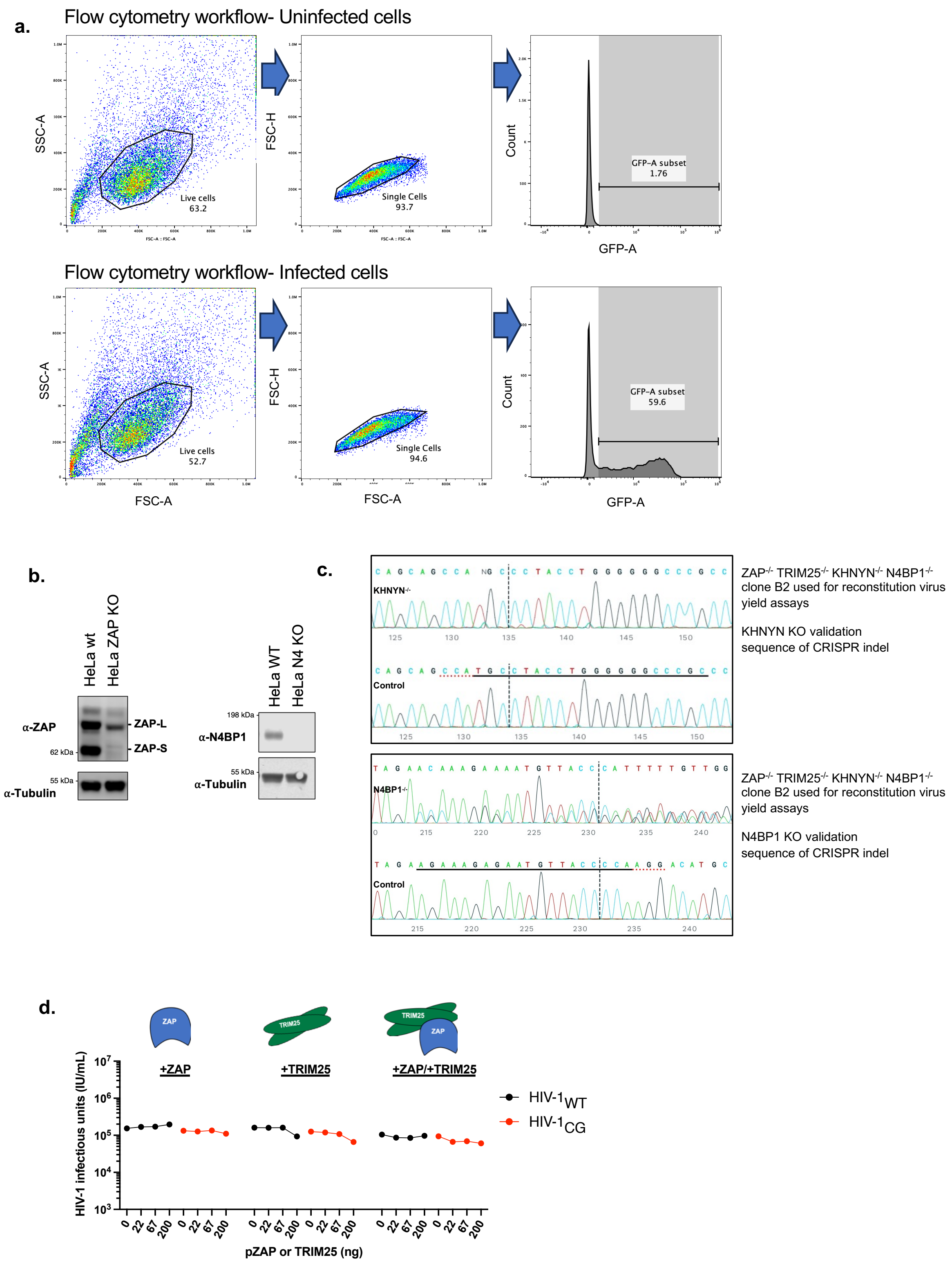
a.
Flow cytometry workflow- Uninfected cells
SSC-A
FSC-H
Count
Live cells
63.2
Single Cells
93.7
GFP-A subset
1.76
GFP-A
Flow cytometry workflow- Infected cells
SSC-A
FSC-H
Count
Live cells
52.7
Single Cells
94.6
GFP-A subset
59.6
FSC-A
FSC-A
GFP-A
b.
HeLa wt
HeLa ZAP KO
α-ZAP
ZAP-L
ZAP-S
62 kDa
55 kDa
α-Tubulin
HeLa WT
HeLa N4 KO
198 kDa
α-N4BP1
55 kDa
α-Tubulin
c.
KHNYN-/-
Control
125
130
135
140
145
150
ZAP-/- TRIM25-/- KHNYN-/- N4BP1-/-
clone B2 used for reconstitution virus yield assays
KHNYN KO validation
sequence of CRISPR indel
N4BP1-/-
Control
215
220
225
230
235
240
ZAP-/- TRIM25-/- KHNYN-/- N4BP1-/-
clone B2 used for reconstitution virus yield assays
N4BP1 KO validation
sequence of CRISPR indel
d.
ZAP
TRIM25
TRIM25
ZAP
+ZAP
+TRIM25
+ZAP/+TRIM25
HIV-1 infectious units (IU/mL)
10^7
10^6
10^5
10^4
10^3
0 22 67 200 0 22 67 200 0 22 67 200 0 22 67 200 0 22 67 200 0 22 67 200
pZAP or TRIM25 (ng)
HIV-1WT
HIV-1CG

Supplementary Figure 1. Knockout validation and KHNYN dependence of reconstitution assay

a. Measurement of infectious virus yield by enumeration of infected (GFP-positive) cells using flow cytometry. Numbers indicte the percentage of cells falling within each gate FSC = forward scatter SSC= side scatter. This workflow is utilized for Figures: 1a-h, 1k-n, 2b-c, 3b-c, 5d-e, 6c, 6f-g, 7b, 7e and Supplementary Figures: 1c, 5b-c, 6a, 6f.

b. Western blot analysis of ZAP and N4PB1 expression in HeLa KO cell lines

c. Analysis of KHNYN and N4BP1 loci targeted by the respective sgRNA following CRISPR knockout.

d. Infectious virus yield in the 293T ZAP$^{-/-}$ TRIM25$^{-/-}$ KHNYN$^{-/-}$ N4BP1$^{-/-}$ reconstitution virus yield from cells transfected with plasmid expressing ZAP or TRIM25 alone or in combination (varying ng amounts of plasmid) in the absence of KHNYN or N4BP1 expression.

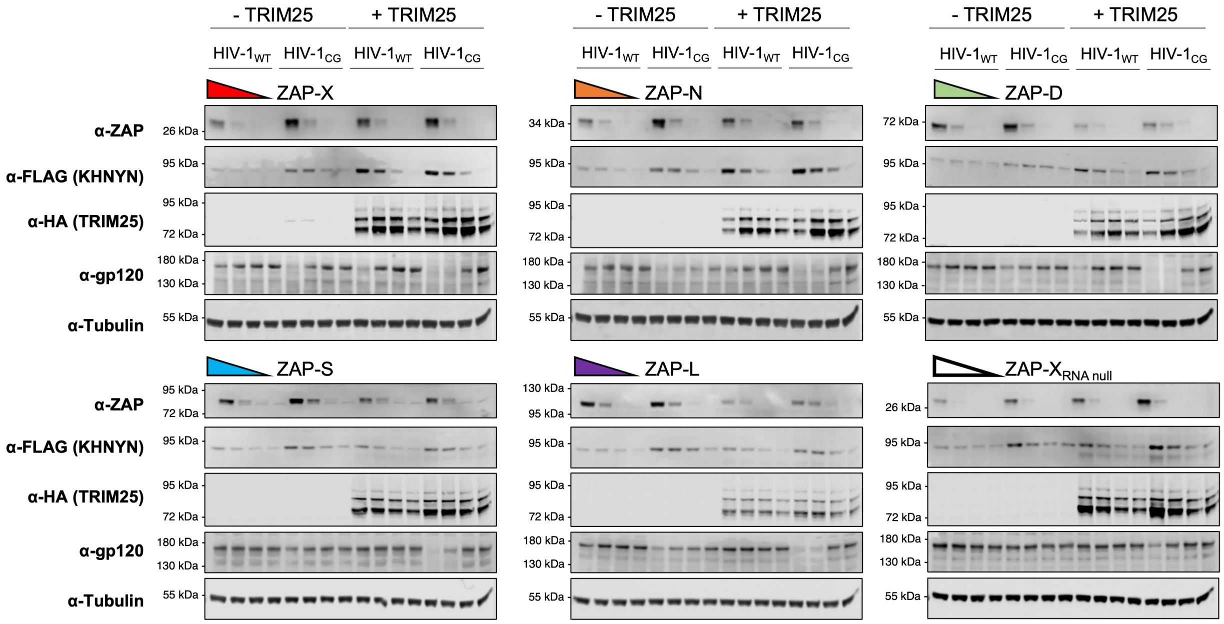

Supplementary Figure 2. Protein expression in ZAP antiviral activity reconstitution assays

Lysates from 293T ZAP$^{-/-}$ TRIM25$^{-/-}$ KHNYN$^{-/-}$ N4BP1$^{-/-}$ cells, transfected as in the reconstitution assays in **Fig 2b,c**, analyzed by western blotting and probing with antibodies against ZAP (for all ZAP-3xHA constructs), KHNYN-FLAG, TRIM25-3xHA, HIV-1 gp120, and Tubulin.

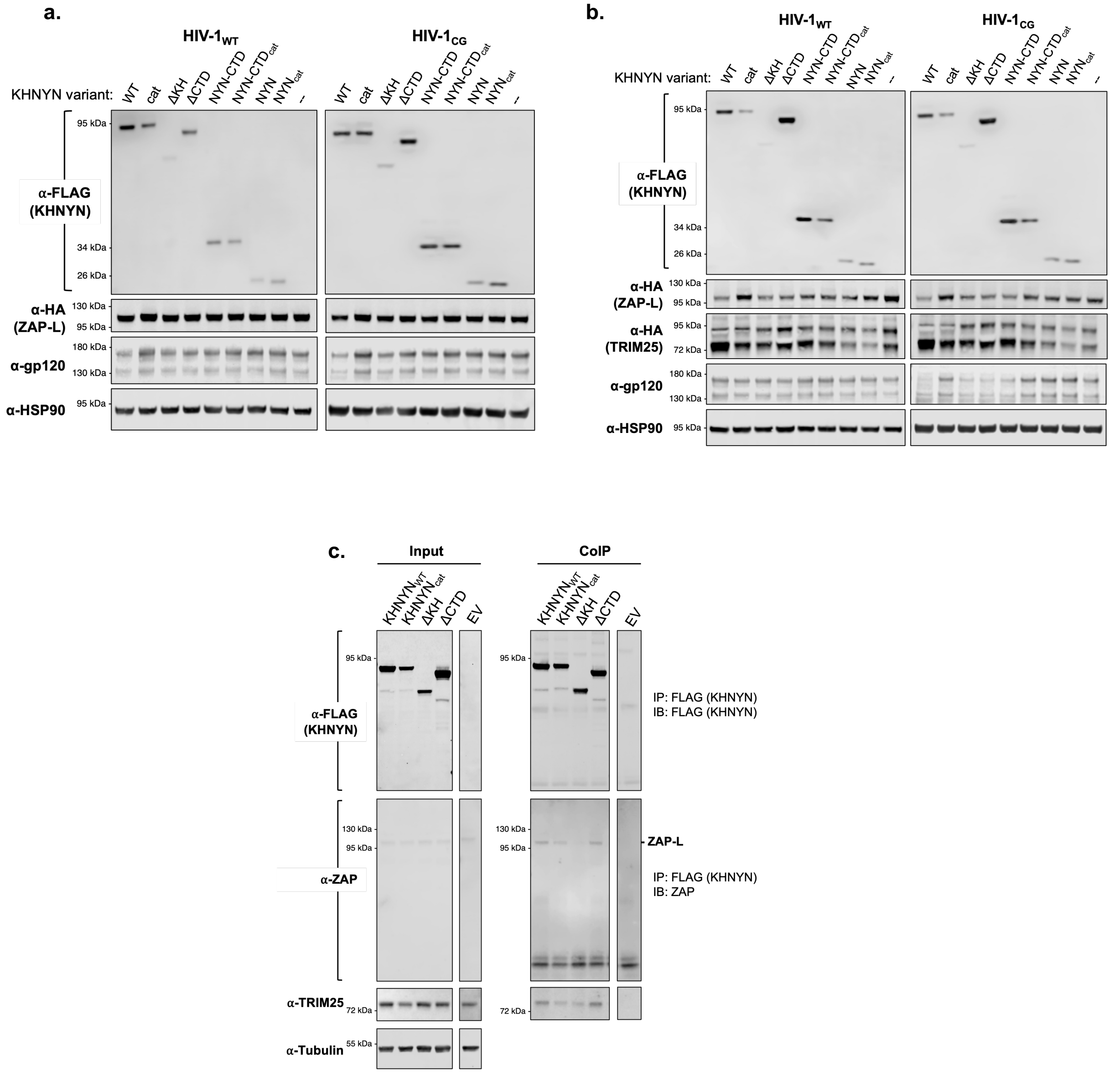

Supplementary Figure 3. Protein expression from KHNYN reconstitution virus yield assays

a, b. Lysates from 293T ZAP$^{-/-}$ TRIM25$^{-/-}$ KHNYN$^{-/-}$ N4BP1$^{-/-}$ cells, transfected as in the reconstitution assays in Fig 3b (**a**) and Fig 3c (**b**), analyzed by western blotting and probing with antibodies against KHNYN-FLAG, ZAP-L-3xHA, TRIM25-3xHA, HIV-1 gp120, and HSP90.

c. Coimmunoprecipitation of endogenous TRIM25 and ZAP-L with KHNYN-FLAG following transfection of 293T KHNYN$^{-/-}$ N4BP1$^{-/-}$ cells with plasmids expressing the indicated KHNYN-FLAG constructs. Proteins were immunoprecipitated from cell lysates with an anti-FLAG antibody and subjected to western blot analyses.

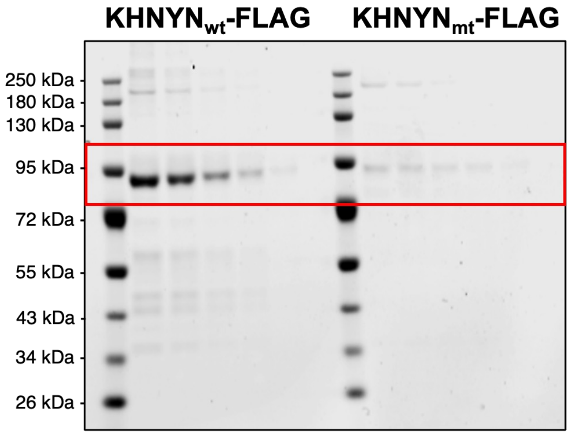

Supplementary Figure 4. SDS-PAGE analysis of proteins used for nuclease assays. Purified, recombinant $KHNYN_{WT}$-FLAG and $KHNYN_{cat}$-FLAG proteins visualized with Coomassie blue stain.

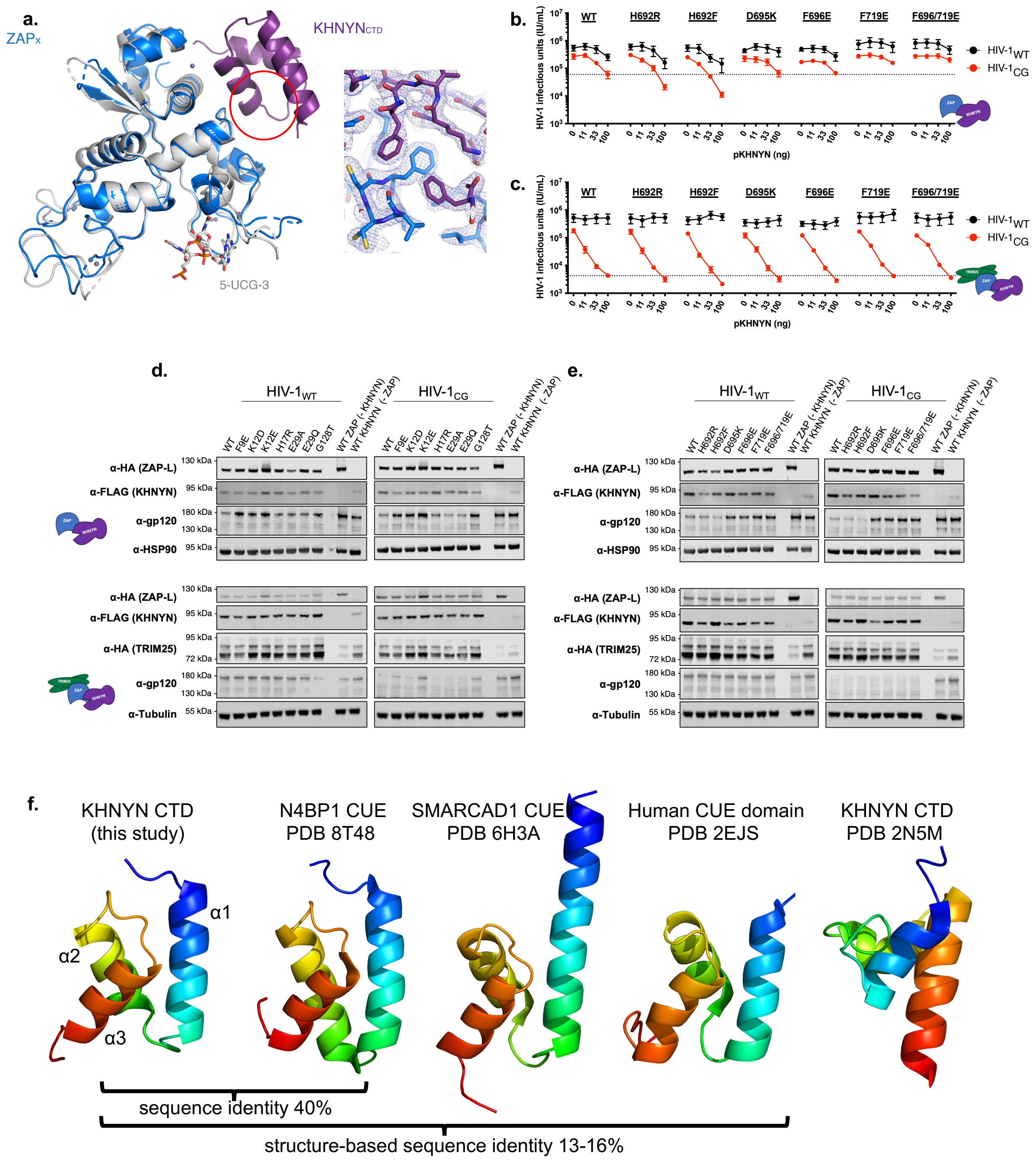
a.
ZAPx
KHNYNCTD
5-UCG-3
b.
WT
H692R
H692F
D695K
F696E
F719E
F696/719E
HIV-1 infectious units (IU/mL)
pKHNYN (ng)
HIV-1WT
HIV-1CG
c.
d.
HIV-1WT
HIV-1CG
WT
F9E
K12D
K12E
H17R
E29A
E29Q
G128T
WT ZAP (- KHNYN)
WT KHNYN (- ZAP)
α-HA (ZAP-L)
α-FLAG (KHNYN)
α-gp120
α-HSP90
α-HA (TRIM25)
α-Tubulin
130 kDa
95 kDa
180 kDa
72 kDa
55 kDa
e.
f.
KHNYN CTD
(this study)
N4BP1 CUE
PDB 8T48
SMARCAD1 CUE
PDB 6H3A
Human CUE domain
PDB 2EJS
KHNYN CTD
PDB 2N5M
α1
α2
α3
sequence identity 40%
structure-based sequence identity 13-16%

Supplementary Figure 5. Structure-function analysis of ZAP-X–KHNYN$_{CTD}$ complex.
a. Overlay of the ZAP-X–RNA complex structure (gray, PDB 6UEI) and the ZAP-X–KHNYN$_{CTD}$ complex (blue ZAP-X, purple KHNYN$_{CTD}$). ZAP-X is virtually identical in the two structures (Cα-atom RMSD 1.06 Å). To the right is an image of electron density for the hydrophobic region in the RBD-CTD interface (2Fo-Fc density contoured at 1.2σ).
b. Reconstitution experiments in which increasing amounts of plasmids (ng) expressing WT and point mutant KHNYN proteins informed by the crystal structure were co-transfected with a constant amount (150 ng) of ZAP expression plasmid in 293T ZAP$^{-/-}$ TRIM25$^{-/-}$ KHNYN$^{-/-}$ N4BP1$^{-/-}$ cells. Effects on HIV-1$_{WT}$ and HIV-1$_{CG}$ virus yield was measured as infectious units (IU/ml). Error bars representative of three biological replicates.
c. Same as **b**. except that 75 ng TRIM25 expression plasmid was also transfected.
d, e. Lysates from 293T ZAP$^{-/-}$ TRIM25$^{-/-}$ KHNYN$^{-/-}$ N4BP1$^{-/-}$ cells, transfected as in Fig 5e,f. (**d**) or Fig S5b,c (**e**) were analyzed by western blotting probing with antibodies against KHNYN-FLAG, ZAP-L-3xHA, TRIM25-3xHA, HIV-1 gp120, and HSP90.
f. Comparison of KHNYN$_{CTD}$ structure determined in this study (9BGL) with homologs from the PDB (8T48, 6H3A, 2EJS) and with the previously published solution structure for KHNYN$_{CTD}$ (2N5M). The published CTD solution structure (2N5M, right) has a different topology and could not be superimposed on the other structures, which are shown in identical views.

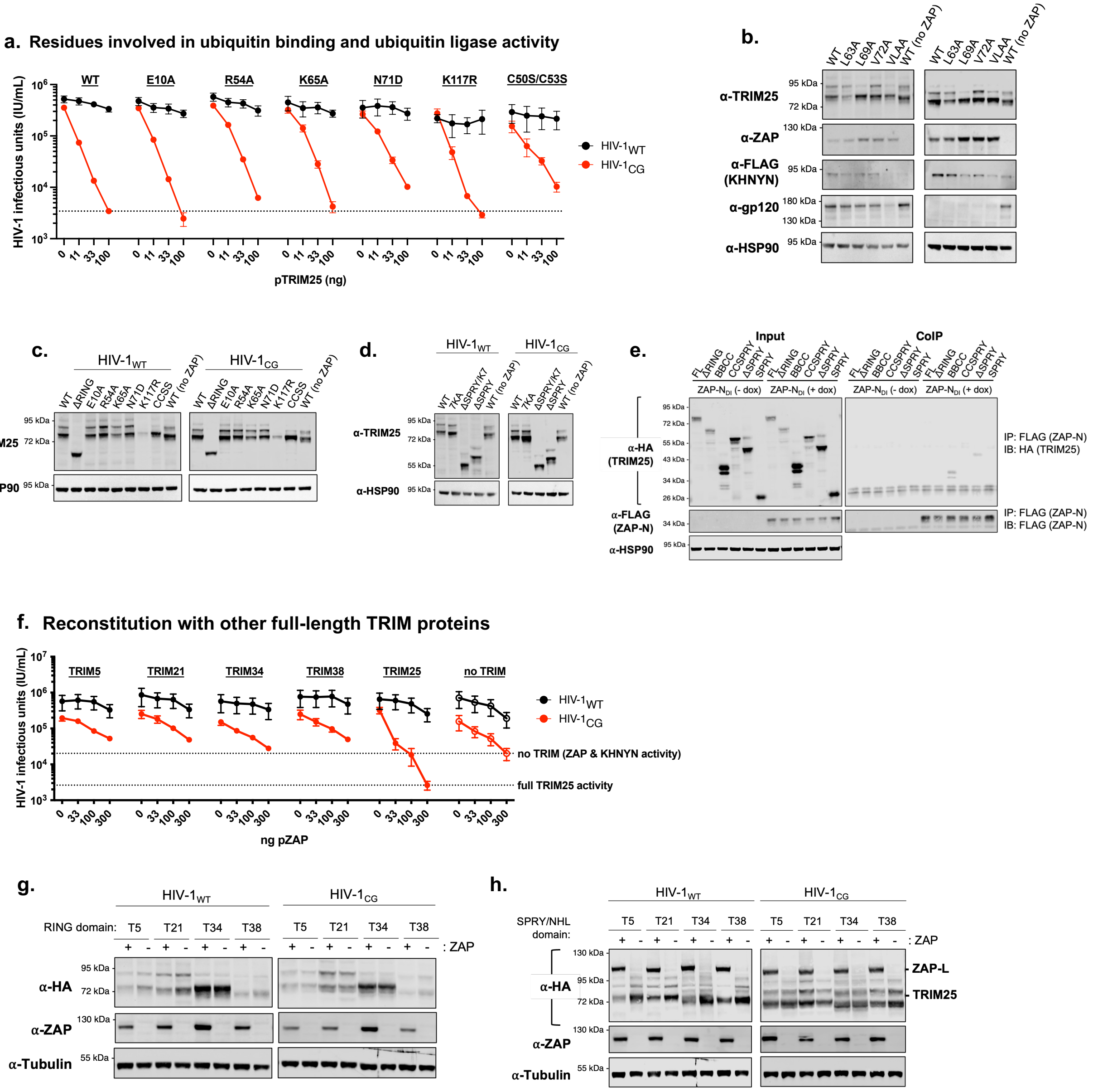

a. Residues involved in ubiquitin binding and ubiquitin ligase activity
WT
E10A
R54A
K65A
N71D
K117R
C50S/C53S
HIV-1 infectious units (IU/mL)
HIV-1WT
HIV-1CG
pTRIM25 (ng)
0 11 33 100
b.
WT L63A L69A V72A VLAA WT (no ZAP)
α-TRIM25
α-ZAP
α-FLAG (KHNYN)
α-gp120
α-HSP90
95 kDa
72 kDa
130 kDa
180 kDa
c.
HIV-1WT
HIV-1CG
WT ΔRING E10A R54A K65A N71D K117R CCSS WT (no ZAP)
α-TRIM25
α-HSP90
d.
WT 7KA ΔSPRY/K7 ΔSPRY WT (no ZAP)
α-TRIM25
α-HSP90
55 kDa
e.
Input
CoIP
FL ΔRING BBCC CCSPRY ΔSPRY SPRY
ZAP-NDI (- dox)
ZAP-NDI (+ dox)
α-HA (TRIM25)
α-FLAG (ZAP-N)
α-HSP90
IP: FLAG (ZAP-N)
IB: HA (TRIM25)
IP: FLAG (ZAP-N)
IB: FLAG (ZAP-N)
95 kDa
72 kDa
55 kDa
43 kDa
34 kDa
26 kDa
f. Reconstitution with other full-length TRIM proteins
TRIM5
TRIM21
TRIM34
TRIM38
TRIM25
no TRIM
HIV-1 infectious units (IU/mL)
HIV-1WT
HIV-1CG
no TRIM (ZAP & KHNYN activity)
full TRIM25 activity
0 33 100 300
ng pZAP
g.
HIV-1WT
HIV-1CG
RING domain:
T5 T21 T34 T38
: ZAP
α-HA
α-ZAP
α-Tubulin
h.
HIV-1WT
HIV-1CG
SPRY/NHL domain:
T5 T21 T34 T38
: ZAP
α-HA
α-ZAP
α-Tubulin
ZAP-L
TRIM25

Supplementary Figure 6. Additional molecular determinants of TRIM25 function.
a. Reconstitution experiments in which increasing amounts of plasmids (ng) expressing WT and point mutant TRIM25 were co transfected with a constant amount of plasmids expressing KHNYN (75 ng) and ZAP-L (150 ng). WT = Wildtype. Antiviral effects were measured against HIV-1_{WT} (black) and HIV-1_{CG} (red). Wildtype TRIM25 was compared with a point mutants at residues involved in ubiquitin binding and ubiquitin ligase activity in the RING domain (E10A, R54A, K65A, N71D, K117R, CCSS). Effects on HIV-1_{WT} (black) and HIV-1_{CG} (red) virus yield was measured as infectious units (IU/ml). Error bars represent three biological replicates.
b. Lysates from 293T ZAP$^{-/-}$ TRIM25$^{-/-}$ KHNYN$^{-/-}$ N4BP1$^{-/-}$ cells, transfected as in **Fig 6b** were analyzed by western blotting probing with antibodies against KHNYN-FLAG, ZAP-L-3xHA, TRIM25-3xHA, HIV-1 gp120, and HSP90.
c. Lysates from 293T ZAP$^{-/-}$ TRIM25$^{-/-}$ KHNYN$^{-/-}$ N4BP1$^{-/-}$ cells, transfected as in **Fig S6a** were analyzed by western blotting probing with antibodies against TRIM25-3xHA and HSP90.
d. Lysates from 293T ZAP$^{-/-}$ TRIM25$^{-/-}$ KHNYN$^{-/-}$ N4BP1$^{-/-}$ cells, transfected as in **Fig 6c** were analyzed by western blotting probing with antibodies against TRIM25-3xHA and HSP90.
e. Coimmunoprecipitation of ZAP-N-FLAG and TRIM25 in 293T ZAP$^{-/-}$ TRIM25$^{-/-}$ KHNYN$^{-/-}$ N4BP1$^{-/-}$ cells transfected with plasmids expressing TRIM25 deletion mutants and stably expressing doxycycline inducible ZAP-N-FLAG (RNA binding domain only). Proteins were immunoprecipitated from cell lysates with anti-FLAG antibody and subjected to western blot analyses.
f. Reconstitution experiments in which increasing amounts of plasmids (ng) expressing the indicated full length TRIM proteins were co transfected with a constant amount of plasmids expressing KHNYN (75 ng) and ZAP-L (150 ng). Effects on HIV-1_{WT} (black) and HIV-1_{CG} (red) virus yield was measured as infectious units (IU/ml). Error bars represent three biological replicates.
g. Lysates from 293T ZAP$^{-/-}$ TRIM25$^{-/-}$ KHNYN$^{-/-}$ N4BP1$^{-/-}$ cells, transfected as in **Fig 6f** were analyzed by western blotting probing with antibodies against TRIM25-3xHA, ZAP-L and Tubulin.
h. Lysates from 293T ZAP$^{-/-}$ TRIM25$^{-/-}$ KHNYN$^{-/-}$ N4BP1$^{-/-}$ cells, transfected as in **Fig 6g** were analyzed by western blotting probing with antibodies against HA (TRIM25-3xHA, ZAP-3xHA) ZAP and Tubulin.

Supplementary Table 1. Plasmid constructs used in this study

Vector back	gene	variant	tag	resistance	cloning method	Notes
pCR3.1	ZAP	aa 1-227 (X)	3xHA	--	cut pCR3.1, gibson insert in	crystallization domain
pCR3.1	ZAP	aa 1-254 (N)	3xHA	--	cut pCR3.1, gibson insert in	slightly larger N-terminus
pCR3.1	ZAP	aa 1-511 (D)	3xHA	--	cut pCR3.1, gibson insert in	includes disordered domain following N-term ZnFs
pCR3.1	ZAP	aa 1-699 (S)	3xHA	--	cut pCR3.1, gibson insert in	naturally occuring isoform, ZAP-S
pCR3.1	ZAP	aa 1-902 (L)	3xHA	--	cut pCR3.1, gibson insert in	full length, ZAP-L
pCR3.1	ZAP	aa 1-227 (X), RNA-null	3xHA	--	cut pCR3.1, gibson insert in	aa 74-76 RRK>AAA, eliminates RNA binding
pCR3.1	ZAP	aa 1-902 (L), RNA-null	3xHA	--	cut pCR3.1, gibson insert in	aa 74-76 RRK>AAA, eliminates RNA binding
pCR3.1	ZAP	aa 255-902, deleted RNA bindir	3xHA	--	cut pCR3.1, gibson insert in	ZAP-L with RNA binding domain deleted entirely
LKO	ZAP	aa 1-254 (N)	3xHA	puro	cut LKO, gibson insert in	
LKO	ZAP	aa 1-902 (L)	3xHA	puro	cut LKO, gibson insert in	
LKO	ZAP	aa 1-254 (N)	Flag	puro	cut LKO, gibson insert in	
LKO	ZAP	aa 1-902 (L)	Flag	puro	cut LKO, gibson insert in	
pCR3.1	ZAP	ZAP-L, F9E	3xHA	--	site directed mutagenesis	mutants for testing of ZAP-N/KHNYN CTD xtal struc
pCR3.1	ZAP	ZAP-L, K12D	3xHA	--	site directed mutagenesis	mutants for testing of ZAP-N/KHNYN CTD xtal struc
pCR3.1	ZAP	ZAP-L, K12E	3xHA	--	site directed mutagenesis	mutants for testing of ZAP-N/KHNYN CTD xtal struc
pCR3.1	ZAP	ZAP-L, H17R	3xHA	--	site directed mutagenesis	mutants for testing of ZAP-N/KHNYN CTD xtal struc
pCR3.1	ZAP	ZAP-L, E29A	3xHA	--	site directed mutagenesis	mutants for testing of ZAP-N/KHNYN CTD xtal struc
pCR3.1	ZAP	ZAP-L, E29Q	3xHA	--	site directed mutagenesis	mutants for testing of ZAP-N/KHNYN CTD xtal struc
pCR3.1	ZAP	ZAP-L, G128T	3xHA	--	site directed mutagenesis	mutants for testing of ZAP-N/KHNYN CTD xtal struc
LKO	KHNYN	full length- KHNYN-2 WT	Flag	puro	from Matt	wildtype KHNYN-2
pCR3.1	KHNYN	full length- KHNYN-2 WT	Flag	--	cut pCR3.1, gibson insert in	wildtype KHNYN-2
pCR3.1	KHNYN	full length- KHNYN-2 DDAA	Flag	--	site directed mutagenesis	catalytic mutant D565,D566A (DDAA)
pCR3.1	KHNYN	deleted KH	Flag	--	cut pCR3.1, gibson insert in	deleted KH domain (aa 12-197 deleted)
pCR3.1	KHNYN	deleted CTD	Flag	--	cut pCR3.1, gibson insert in	deleted CTD domain (aa 667-719 deleted)
pCR3.1	KHNYN	KH domain alone	Flag	--	cut pCR3.1, gibson insert in	KH domain alone (aa 12-197)
pCR3.1	KHNYN	NYN-CTD (NC) WT	Flag	--	cut pCR3.1, gibson insert in	aa 451-719, WT
pCR3.1	KHNYN	NYN-CTD DDAA	Flag	--	cut pCR3.1, gibson insert in	aa 451-719, DDAA catalytic mt
pCR3.1	KHNYN	NYN	Flag	--	cut pCR3.1, gibson insert in	aa 451-634, wt
pCR3.1	KHNYN	NYN DDAA	Flag	--	cut pCR3.1, gibson insert in	aa 451-634, DDAA catalytic mt
pCR3.1	KHNYN	Y497A	Flag	--	site directed mutagenesis	mutation of possible phosphorylation site
pCR3.1	KHNYN	K656A	Flag	--	site directed mutagenesis	mutation of possible ubiquitylation site
pCR3.1	KHNYN	H692F	Flag	--	site directed mutagenesis	mutants for testing of ZAP-N/KHNYN CTD xtal struc
pCR3.1	KHNYN	H692R	Flag	--	site directed mutagenesis	mutants for testing of ZAP-N/KHNYN CTD xtal struc
pCR3.1	KHNYN	D695K	Flag	--	site directed mutagenesis	mutants for testing of ZAP-N/KHNYN CTD xtal struc
pCR3.1	KHNYN	F696E	Flag	--	site directed mutagenesis	mutants for testing of ZAP-N/KHNYN CTD xtal struc
pCR3.1	KHNYN	F719E	Flag	--	site directed mutagenesis	mutants for testing of ZAP-N/KHNYN CTD xtal struc
pCR3.1	KHNYN	F696, 719E	Flag	--	site directed mutagenesis	mutants for testing of ZAP-N/KHNYN CTD xtal struc
pCR3.1	KHNYN	aa 1-718	Flag	--	cut pCR3.1, gibson insert in	mutants for testing of ZAP-N/KHNYN CTD xtal struc
pCR3.1	TRIM25	WT	3xHA	--	cut pCR3.1, gibson insert in	aa 1-630
pCR3.1	TRIM25	deleted RING_1	3xHA	--	cut pCR3.1, gibson insert in	aa 83-630, deleted RING domain
pCR3.1	TRIM25	deleted RING_2	3xHA	--	cut pCR3.1, gibson insert in	aa 102-630, deleted RING (and space between RIN
pCR3.1	TRIM25	RING	3xHA	--	cut pCR3.1, gibson insert in	aa 1-82, RING domain alone
pCR3.1	TRIM25	RBB	3xHA	--	cut pCR3.1, gibson insert in	aa 1-189, RING and Bbox domains
pCR3.1	TRIM25	BBCC_1	3xHA	--	cut pCR3.1, gibson insert in	aa 83-379, Bbox and Coiled coil (with space betweer
pCR3.1	TRIM25	BBCC_2	3xHA	--	cut pCR3.1, gibson insert in	aa 102-379, Bbox and Coiled coil
pCR3.1	TRIM25	CC	3xHA	--	cut pCR3.1, gibson insert in	aa 189-379, Coiled coil
pCR3.1	TRIM25	CCSPRY	3xHA	--	cut pCR3.1, gibson insert in	aa 189-630, Coiled coil and SPRY
pCR3.1	TRIM25	SPRY	3xHA	--	cut pCR3.1, gibson insert in	aa 434-630, SPRY domain alone
pCR3.1	TRIM25	deleted 7K and SPRY	3xHA	--	cut pCR3.1, gibson insert in	aa 1-379, deleted 7 lysines and SPRY domain
pCR3.1	TRIM25	deleted SPRY	3xHA	--	cut pCR3.1, gibson insert in	aa 1-410, deleted SPRY domain
pCR3.1	TRIM25	7KA	3xHA	--	from Matt	7 lysines mutated to alanine (aa 381,382, 385, 388, 3
pCR3.1	TRIM25	E10A	3xHA	--	site directed mutagenesis	mutatnts in RING domain to test TRIM25 function
pCR3.1	TRIM25	R54A	3xHA	--	site directed mutagenesis	mutatnts in RING domain to test TRIM25 function
pCR3.1	TRIM25	L63A	3xHA	--	site directed mutagenesis	mutatnts in RING domain to test TRIM25 function
pCR3.1	TRIM25	K65A	3xHA	--	site directed mutagenesis	mutatnts in RING domain to test TRIM25 function
pCR3.1	TRIM25	L69A	3xHA	--	site directed mutagenesis	mutatnts in RING domain to test TRIM25 function
pCR3.1	TRIM25	N71D	3xHA	--	site directed mutagenesis	mutatnts in RING domain to test TRIM25 function
pCR3.1	TRIM25	V72A	3xHA	--	site directed mutagenesis	mutatnts in RING domain to test TRIM25 function
pCR3.1	TRIM25	V68, L69A	3xHA	--	site directed mutagenesis	mutatnts in RING domain to test TRIM25 function
pCR3.1	TRIM5	full length TRIM5	3xHA	--	cut pCR3.1, gibson insert in	test various TRIMs in reconstitution assay
pCR3.1	TRIM21	full length TRIM21	3xHA	--	cut pCR3.1, gibson insert in	test various TRIMs in reconstitution assay
pCR3.1	TRIM34	full length TRIM34	3xHA	--	cut pCR3.1, gibson insert in	test various TRIMs in reconstitution assay
pCR3.1	TRIM38	full length TRIM38	3xHA	--	cut pCR3.1, gibson insert in	test various TRIMs in reconstitution assay
pCR3.1	TRIM25 chimer	TRIM25 w/ TRIM5 RING	3xHA	--	cut pCR3.1, gibson insert in	TRIM25 chimera with various RING domains
pCR3.1	TRIM25 chimer	TRIM25 w/ TRIM21 RING	3xHA	--	cut pCR3.1, gibson insert in	TRIM25 chimera with various RING domains
pCR3.1	TRIM25 chimer	TRIM25 w/ TRIM34 RING	3xHA	--	cut pCR3.1, gibson insert in	TRIM25 chimera with various RING domains
pCR3.1	TRIM25 chimer	TRIM25 w/ TRIM38 RING	3xHA	--	cut pCR3.1, gibson insert in	TRIM25 chimera with various RING domains
pCR3.1	TRIM25 chimer	TRIM25 w/ TRIM5 SPRY	3xHA	--	cut pCR3.1, gibson insert in	TRIM25 chimera with various SPRY domains
pCR3.1	TRIM25 chimer	TRIM25 w/ TRIM21 SPRY	3xHA	--	cut pCR3.1, gibson insert in	TRIM25 chimera with various SPRY domains
pCR3.1	TRIM25 chimer	TRIM25 w/ TRIM34 SPRY	3xHA	--	cut pCR3.1, gibson insert in	TRIM25 chimera with various SPRY domains
pCR3.1	TRIM25 chimer	TRIM25 w/ TRIM38 SPRY	3xHA	--	cut pCR3.1, gibson insert in	TRIM25 chimera with various SPRY domains
pCR3.1	TRIM25 chimer	TRIM25 w/ ZAP-N as SPRY, cl	3xHA	--	cut pCR3.1, gibson insert in	TRIM25 chimera with ZAP N-terminus in place of SP
pCR3.1	ZAP/KHNYN fu	ZAP X- KHNYN NYN	Flag	--	cut pCR3.1, gibson inserts in	fusion A- KHNYN NYN
pCR3.1	ZAP/KHNYN fu	ZAP X- KHNYN NYN DDAA	Flag	--	cut pCR3.1, gibson inserts in	fusion A- KHNYN NYN

Supplementary Table 2. Summary of crystallography

**Diffraction data**	**ZAP_X-$KHNYN_{CTD}$**
PDB ID	9BGL
Space group	$P2_12_12_1$
Unit cell (Å)	36.214, 88.001, 94.547
Wavelength (Å)	0.97875
d_{min} (Å)	2.30 (2.30-2.34)[1]
R_{sym}[2] (%)	6.5 (54.8)
$CC_{½}$	1.00 (0.90)
<I/σI>	27.0 (3.7)
Unique reflections	14,164
Completeness (%)	100 (100)
Redundancy	7.1 (7.2)
**Refinement**	
Data range (Å)	44-2.30
# reflections	13,430
R_{work} [3]	0.206
R_{free} [4]	0.245
Amino acid residues	265
Protein atoms	2075
Waters	131
R.m.s.d.[5]	
Bonds (Å)	0.007
Angles (°)	0.85
Ramachandran	
Favored (%)	96.46
Allowed (%)	3.54
Outliers (%)	0
MolProbity clash score	2.16

[1]Statistics for the outermost shell of data are in parentheses.
[2]$R_{sym} = \Sigma_h\Sigma_j \mid I_{hj} - \langle I_h \rangle \mid / \Sigma_h\Sigma_j I_{hj}$, where I_{hj} is the intensity of observation j of reflection h and $\langle I_h \rangle$ is the mean intensity for multiply recorded reflections.
[3]R-factor = $\Sigma_h \mid |F_o| - |F_c| \mid / \Sigma_h|F_o|$, where F_o and F_c are the observed and calculated structure factor amplitudes for reflection h.
[4]R_{free} was calculated for a random 5% of reflections that were excluded from refinement.
[5]Root mean square deviation of bond lengths and bond angles from target values.
