# Supplementary material for: Functional anatomy of zinc finger antiviral protein complexes
Source: Nat Commun. 2024 Dec 30;15:10834. doi: 10.1038/s41467-024-55192-z (PMC11685948; doi:10.1038/s41467-024-55192-z)
Supplement: Supplementary file 3 — Description of Additional Supplementary Files [file 41467_2024_55192_MOESM3_ESM.pdf]

Description of Additional Supplementary Files

Supplementary Data 1: Oligonucleotides used in this study for molecular construction
